# Relationship between maximal oxygen uptake, within-set fatigue and between-set recovery during resistance exercise in resistance-trained men and women

**DOI:** 10.1186/s13102-024-00830-8

**Published:** 2024-02-12

**Authors:** Tommy R. Lundberg, Gustav Larsson, Rasmus Alstermark, Mirko Mandić, Rodrigo Fernandez-Gonzalo

**Affiliations:** 1 Department of Laboratory Medicine, Division of Clinical Physiology, ANA FUTURA, Karolinska Institutet, Stockholm, Huddinge 14152 Sweden; 2 Unit of Clinical Physiology, Karolinska University Hospital, Stockholm, Sweden

**Keywords:** Muscle fatigue, Inter-set rest, Sex differences, Strength training, V̇O_2max_

## Abstract

**Background:**

The primary aim of this study was to examine the relationship between maximal oxygen update (V̇O_2max_) and within-set fatigue and between-set recovery during resistance exercise in men and women.

**Methods:**

We examined the relationship between V̇O_2max_ and various indices of fatigue and recovery during parallel squats (3 sets, 90 s rest, 70% of 1RM to failure) and isokinetic knee extensions (3 × 10 maximal repetitions at 60 deg/s, 45 s rest) in 28 (age 27.0 ± 3.6 years) resistance-trained subjects (14 men and 14 women). We also examined whether there were sex differences in within-set fatigue and between-set recovery.

**Results:**

V̇O_2max_ was weakly related to recovery and fatigue in both men and women (range of P-values for V̇O_2max_ as a covariate; 0.312–0.998, range of R-values, 0.005–0.604). There were no differences between the sexes in fatigue within a set for the squat, but men showed less within-set fatigue than women in the first set of the isokinetic knee extension exercise (~ 8% torque loss difference, main effect of sex *P* = 0.034). Regarding recovery between sets, men showed greater relative peak power (*P* = 0.016) and peak torque (*P* = 0.034) loss between sets in both exercises, respectively, compared to women. Women also tended to complete more repetitions than men (main effect of sex, *P* = 0.057). Loss of peak torque between sets in knee extension was evident in both absolute and relative (%) values in men but not in women.

**Conclusions:**

Our study suggests that aerobic capacity is weakly associated with within-set fatigue and between-set recovery in resistance training in both men and women. Women and men show comparable levels of within-set fatigue in the multi-joint squat, but women show more within-set fatigue during the single-joint isokinetic knee extension compared with men. In contrast, women recover better than men between sets in both exercises.

**Supplementary Information:**

The online version contains supplementary material available at 10.1186/s13102-024-00830-8.

## Background

Resistance training is a cornerstone of athletic conditioning and physical fitness. Effective resistance training incorporates training principles such as overload and progression, and key program variables include intensity, volume, and strategic manipulation of rest periods between sets (often ranging from 30 s to 5 min) [1]. While a short rest period is more time efficient and allows for more exercises or sets to be performed overall within a training session of same duration, it can also negatively affect performance in the next set due to residual fatigue [2]. Furthermore, resistance exercise with short (1 min) rest periods between sets resulted in a lower myofibrillar protein synthesis during the early post-exercise recovery period compared with longer (5 min) rest periods, possibly through impaired activation of intracellular signalling [3]. Thus, the rest interval between sets influences acute performance, metabolic stress, and anabolic response, and may therefore affect the muscular adaptations during chronic exercise [4]. Finding the optimal length of rest between sets, ideally on an individual basis and depending on the goal of the training, is therefore important for resistance training practitioners who wish to maximise the benefits of resistance training.

The interplay between the duration of rest and substrate replenishment becomes clear when considering that ATP supply during high-intensity resistance exercise occurs mainly via anaerobic pathways involving muscle glycogen and phosphocreatine (PCr) as fuel substrates [5]. Regeneration of PCr stores, which has been found to be related to oxygen availability and aerobic capacity [6, 7], is generally considered to be the most important means of restoring immediate performance following strenuous muscular exercise. This is supported by the notion that recovery from repeated sprint and high intensity intermittent exercise is related to PCr resynthesis and aerobic capacity [8, 9]. It is therefore possible that high aerobic capacity allows faster recovery between sets also during resistance exercise, and that the optimization of rest intervals should be dictated not only by individual training goals, but also on an individual’s capacity to restore phosphagen stores between sets.

There is surprisingly little information on the relationship between aerobic capacity and recovery of performance during resistance exercise sessions. Ratamess et al. examined the relationship between maximal oxygen uptake (V̇O_2max_) and acute performance during resistance exercise using 1-, 2-, or 3-minute rest intervals in random order [10]. V̇O_2max_ was found to be negatively correlated with the 1RM bench press and squat performance, but positively correlated with the number of repetitions subjects were able to perform during the squat (5 sets at 75% of 1RM). The authors concluded that V̇O_2max_ was related to lower body resistance exercise performance and hypothesized that individuals with a higher V̇O_2max_ would have a greater ability to maintain performance between sets during lower body exercises.

Overall, there appears to be conflicting evidence as to whether there are sex differences in fatigability and strength recovery during resistance exercise, and the specific program design, exercise selection, and contraction type may partly explain the conflicting findings [11]. Nuzzo et al. reported on the maximum number of repetitions at different percentages of the 1RM and found that sex had little effect on the outcome [12]. However, they emphasized that there is little data on exercises outside of the bench press and leg press. Men have been reported to be more fatigued than women during sustained and intermittent isometric exercise performed at similar relative intensities [11, 13]. Men have also been shown to recover more slowly than women after isometric exercise, possibly due to greater central fatigue in men or, alternatively, better local muscle endurance in women [14–16]. Despite this evidence, Gomes et al. recently reported that women showed greater torque loss after fast isokinetic muscle contractions (300 deg/s), but not in response to contractions performed at slow speeds (60 deg/s) [17]. However, strength recovery was greater in women after exercise at moderate speeds (180 deg/s). There is also a paucity of data examining sex differences in fatigue and recovery following isotonic multi-joint exercise involving several large muscle groups, and to our knowledge no study has examined this in relation to aerobic capacity. This is pertinent to investigate as training with larger muscle mass involvement shows a stronger relationship between V̇O_2max_ and PCr resynthesis than intense training with small muscle mass involvement [8].

With this in mind, we sought to comprehensively investigate the relationship between V̇O_2max_ and within-set fatigue and between-set recovery during resistance exercise in both men and women. We also investigated whether there were sex differences in the fatigue response and recovery pattern between sets. We hypothesised that V̇O_2max_ would be positively related to between-set recovery for the squat, but not for the isolated knee extension involving a smaller muscle mass. We also hypothesised that women would show less fatigue and recover faster than men during both exercises.

## Methods

### General design

Each subject was tested for V̇O_2max_, maximum number of repetitions in squat at 70% of 1RM, peak concentric power in squat, and peak torque production during 3 × 10 repetitions of isokinetic knee extensions. We then examined the relationship between V̇O_2max_ and various indices of within-set fatigue and between-set recovery.

### Subjects

The subjects of the present study (14 men and women) were physically active young adults from the Stockholm area (Table 1). A power calculation performed in GPower showed that we would need a sample size of 23 to detect a Pearson correlation (r-value) of 0.5, which we consider a meaningful relationship, with a power of 80% and an alpha of 5%. Nevertheless, we included a total of 28 subjects to also allow for comparison by sex. Inclusion criteria included resistance training experience (3 sessions per week for at least 3 months prior to the study) and adequate squat exercise technique (assessed by the investigators). A health questionnaire (see Supplementary Questionnaire 1) was completed at the screening stage, but no participant was excluded for health reasons. All subjects provided written informed consent prior to participation. The study was approved by the National Review Authority in Sweden (Ref no. 020-03551).

[Cut]

**Table 1:** Descriptive characteristics and performance results

	Women	Men	All	
Age (yr)	26.1 ± 2.5	27.9 ± 4.4	27.0 ± 3.6	
Height (cm)	166.1 ± 5.5	185.4 ± 6.6*	175.8 ± 11.5	
Weight (kg)	63.9 ± 7.5	89.4 ± 10.0*	76.7 ± 15.6	
HR_max_ (bpm)	184.7 ± 8.8	186.6 ± 8.9	185.6 ± 8.7	
Borgs RPE	17.6 ± 1.3	18.5 ± 1.2	18.1 ± 1.3	
V̇O_2max_ (L/min)	3.0 ± 0.3	4.5 ± 0.4*	3.7 ± 0.8	
V̇O_2max_ (ml/kg/min)	46.9 ± 4.3	50.5 ± 6.4	48.7 ± 5.6	
W_max_	258.8 ± 23.8	341.1 ± 35.3*	300.0 ± 51.3	
1RM (kg)	81.6 ± 18.1	149.1 ± 35.8*	115.4 ± 44.2	
Workload squat (kg)	58.1 ± 12.3	103.9 ± 25.1*	81.0 ± 30.3	

W_max_; maximal workload achieved during the V̇O_2max_ cycling test, 1RM; estimated one repetition maximum in squat exercise. Sex differences were analyzed with independent t-test

*represents *p* < 0.001

### Maximum oxygen uptake (V̇O_2max_)

To measure V̇O_2max_, subjects performed a graded test to volitional fatigue on a cycle ergometer (Lode, Groningen, The Netherlands). An online gas system (Carefusion, Yorba Linda, CA) was used to directly measure breath-by-breath respiratory volume, respiratory flow, and exhaled O_2_ and CO_2_ content (35,36). Subjects were given verbal instructions on how to perform the V̇O_2max_ test before starting the test. The bike was set up according to the subjects’ preferences and a gas analysis mask was placed over the subjects’ mouth and nose. The test began with subjects cycling for 5 min at a resistance of 50 watts. The resistance was then increased by one watt every three seconds until the participant could no longer maintain a cadence of 60–65 rpm. The same protocol was employed for all subjects and the average test duration was 12.5 min. The bicycle was connected to a computer that increased resistance via a pre-determined protocol. Subjects were verbally encouraged by the test leaders until they reached voluntary exhaustion. Heart rate was measured every 15 s. V̇O_2max_ was determined as the highest average oxygen uptake within a 20-second period during the test. A plateau in oxygen uptake despite increased intensity was used as a main criterion for V̇O_2max_. No subsequent verification test was performed. Respiratory Exchange Ratio (RER) > 1.15, a heart rate > 90% of the age-predicted maximum heart rate, and rating of perceived exertion (RPE) of > 17 (Borg scale) collected immediately after exhaustion were used as additional criteria when a plateau was not established. The CV% for V̇O_2max_ assessment in our laboratory is 1.7–2.3%.

### Squat exercise

The Squat protocol included a 1 repetition maximum (1RM) test followed by three sets of as many repetitions as possible at 70% of the 1RM. Mean concentric movement velocity during squats was measured using a linear encoder (SmartCoach Europe AB, Stockholm, Sweden), and peak power values were then calculated based on the movement velocity and weight lifted. The selection of the squat was based on the use of large muscle mass covering several joints. 1RM testing took place 3–7 days after the V̇O_2max_ test. The procedure began with a standardised warm-up protocol including cycling for 5 min on a bicycle with a self-selected power output of 80–100 W. The warm-up was completed with squats using the 20 kg barbell as loading (5 repetitions). After the warm-up, four to six progressively heavier sets (corresponding to 5 reps at 50%, 3 reps at 70%, 1 rep at 80% and 1 rep at 90% of estimated 1RM) were conducted prior to reaching 1RM in subsequent lifts. After the 1RM had been assessed, subjects were allowed to recover for 3 min before commencing the Squat exercise trial at 70% of the 1RM. Three sets were performed at maximal effort, i.e., as many repetitions as possible to failure. Failure was defined on the basis of three criteria: Inability to rise from a deep squat, letting go of the barbell despite verbal encouragement, or performing two consecutive squats with insufficient depth as evidenced by not lowering the greater trochanter below the apex of the patella. We standardised the depth by placing safety bars just below the bar at the participants’ bottom position. The foot position was chosen by the participants themselves, as we wanted to minimise the impact on their technical performance. Between each repetition, participants were allowed to take a breath, but we encouraged them to go back down as quickly as possible. Each set was interspersed by 90 s of rest.

### Isokinetic knee extensions

The knee extension protocol was performed on an isokinetic dynamometer (Biodex System 4 Pro, Medical Systems, Shirley, NY) and consisted of three sets of ten repetitions to examine changes in torque between and within sets. The selection of knee extension was based on the isolated nature of this exercise, and the smaller muscle mass involved. The knee extension test was performed on the right leg of the subjects. The dynamometer was set to a range of motion of 90° from vertical to horizontal with a velocity of 60°/s. Prior to the start of the test, subjects completed a set of ten repetitions at a submaximal effort for familiarisation. During the test, subjects performed ten repetitions at maximum effort (under strong verbal encouragement) for three sets with 45 s rest between sets. The CV% for torque assessment using the Biodex in our laboratory is 3.2–4.0% (at slow angular velocities).

### Within-set fatigue and between-set recovery

Within-set fatigue for peak power in the squat exercise was calculated as the relative difference (in %) between the average peak power of the first two and the last two repetitions in each set. Within-set fatigue for peak torque during the isokinetic knee extension exercise was calculated as the relative difference between repetitions 2–3 and repetitions 9–10 in each set. This was chosen because the first repetition of each set was 5–12% lower than the values obtained in repetitions 2 and 3. Between-set recovery was calculated as the relative difference (in %) in peak power (squat; average peak power within a set) and peak torque (knee extensions; average peak torque within a set) between sets, i.e., set 1 vs. 2, set 2 vs. 3, and set 1 vs. 3.

### Methodological considerations

Subjects were allowed to consume food up until two hours before the tests were performed. Consumption of caffeine was allowed. Subjects were not allowed to do any other strenuous exercise within 48 h prior to performing the tests. If subjects were taking creatine supplements (*n* = 6), they were asked to maintain this supplementation throughout the study period.

### Data analysis

The main statistical analysis aimed to assess whether within-set fatigue and between-set recovery during resistance exercise were related to aerobic capacity (i.e., V̇O_2max_). For this purpose, we used mixed model analyses with set and sex as fixed factors, and V̇O_2max_ as covariate for all fatigue and recovery variables. This allowed us to establish the importance of V̇O_2max_ in the fatigue and recovery indices explored. To further investigate the relationship between V̇O_2max_ and fatigue/recovery, we used Pearson’s correlation coefficient (R-value). As a secondary analysis, we examined possible differences between men and women in within-set fatigue and between-set recovery using mixed model analyses with set and sex as factors. When significant interactions were found, Bonferroni post-hoc corrections were performed. When main effects of sex were detected, we also included the relative weight lifted during the squats in the mixed model to determine whether baseline strength, rather than sex, influenced the outcome. In addition, we used independent t-tests to analyse potential differences between sexes in baseline characteristics, as well as absolute performance outcomes. The significance level was set at 5% (*P* < 0.05). Statistical analyses were performed using Prism 7 for Mac OS X (GraphPad Software, San Diego, CA) and Jamovi (v1.6.23.0). Data are presented as means ± SD or as relative changes in percentage units.

## Results

The descriptive characteristics, together with the results from the V̇O_2max_ and 1RM test, are shown in Table 1. As expected, men had higher (~ 50%) V̇O_2max_ in absolute values and reached a higher (~ 30%) workload during the incremental test than women. Also, men showed higher 1RM squat values compared with women (~ 80%).

### Relationship between V̇O_2max_ and within-set fatigue and between-set recovery

**Fig. 1:**
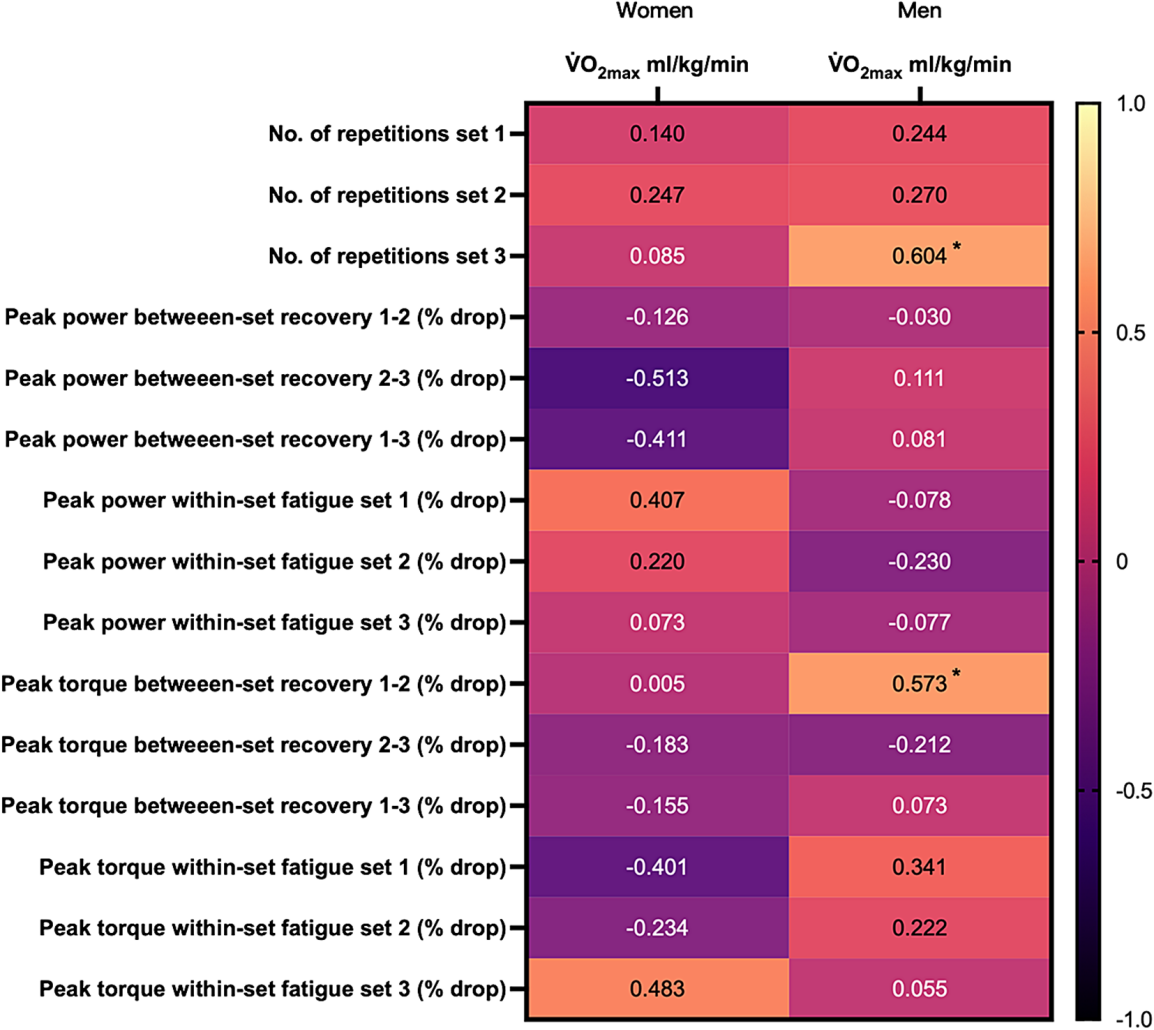
Heatmap showing the correlation level (Pearson’s r) between the V̇O_2max_ and the fatigue and recovery parameters analysed. The lighter the colour of the square, the higher the positive correlation; the darker the colour of the square, the higher the negative correlation (see colour code in the bar to the right). *indicates significant correlation; *P* < 0.05

The mixed model analyses performed on within-set fatigue and between-set recovery for peak power and peak torque, with V̇O_2max_ as covariate, indicated that maximal aerobic capacity did not significantly covary with any of the changes seen in these performance variables (range of P-values for V̇O_2max_ as a covariate; 0.312–0.998). Consistent with this, the correlations between V̇O_2max_ and fatigue and recovery variables were relatively weak for both men and women (Fig. 1). The only significant correlations found occurred in men and were noted between V̇O_2max_ and the number of squat repetitions in set 3 (*r* = 0.604, *P* = 0.022), and V̇O_2max_ and torque recovery between the 1st and the 2nd sets (*r* = 0.573, *P* = 0.032).

### Fatigue and recovery during squat exercise

**Fig. 2:**
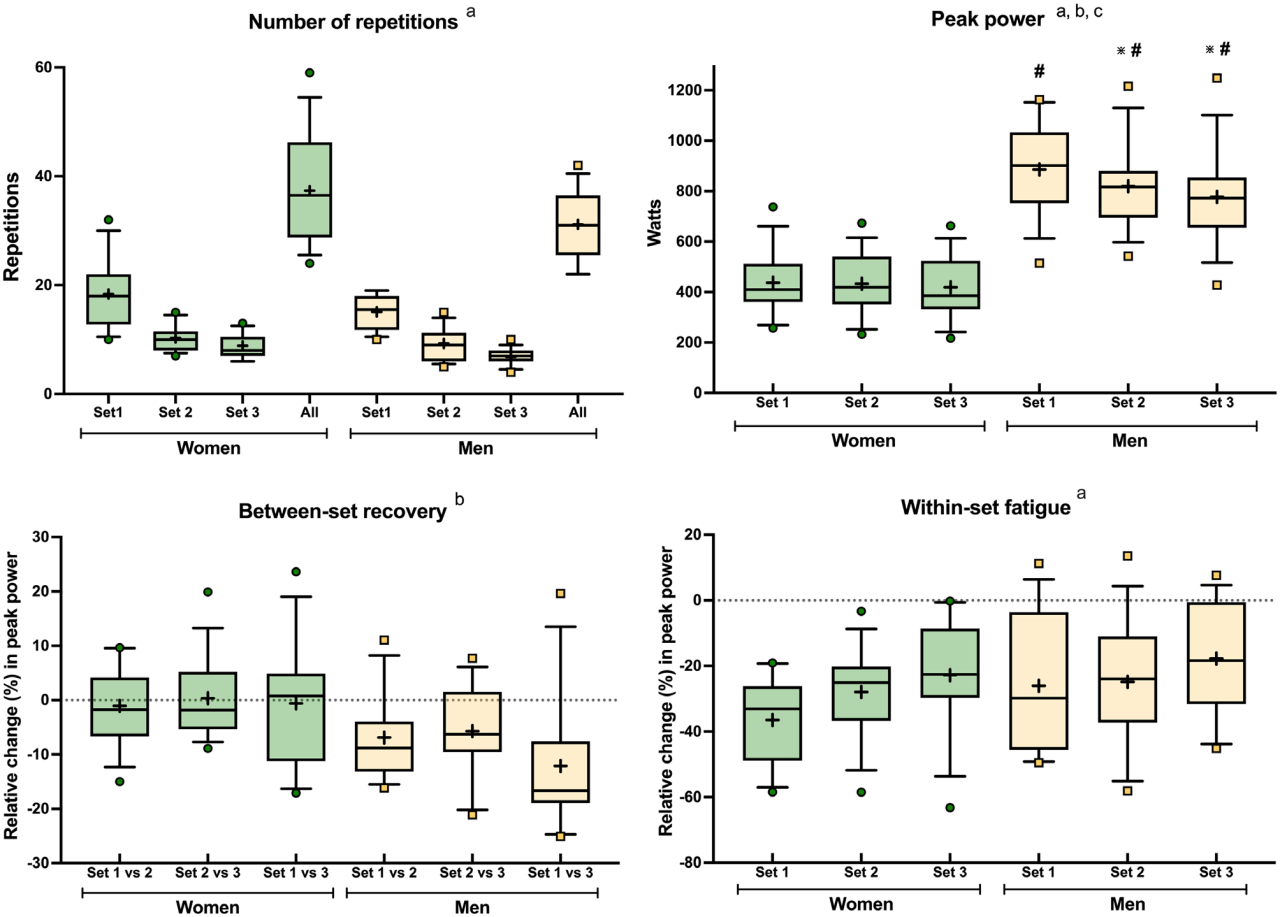
Performance, recovery, and fatigue variables from the squat exercise for both women (green) and men (yellow). The boxes extend from the 25th to the 75th percentile, the line in the middle is the median, and the + symbol is the mean. The whiskers represent the 10th and 90th percentiles. a; main effect of set, b; main effect of sex (*P* = 0.056 if in grey), c; interaction set*sex. Post hocs: *Different with set 1 within a sex, #different to women

The number of repetitions performed in the squat exercise (Fig. 2) decreased in both men and women from set 1 to set 3 (main effect of set, *P* < 0.001). Overall, women showed a tendency to perform more repetitions (approximately 6 more repetitions in total) than men (main effect of sex, *P* = 0.057; difference women vs. men for total number of repetitions using t-test *P* = 0.065). When investigating between-set recovery, men showed greater peak power losses between sets compared with women, both in absolute (interaction set*sex *P* < 0.001; Fig. 2) and relative terms (main effect of sex *P* = 0.016; Fig. 2). In contrast, there were no differences between sexes in within-set fatigue for the squat exercise. Thus, both men and women fatigued more in set 1 (~ 30% power loss) than in sets 2 (~ 25% power loss) and 3 (~ 20% power loss) (main effect of set *P* < 0.001; Fig. 2).

### Fatigue and recovery during isokinetic knee extension exercise

**Fig. 3:**
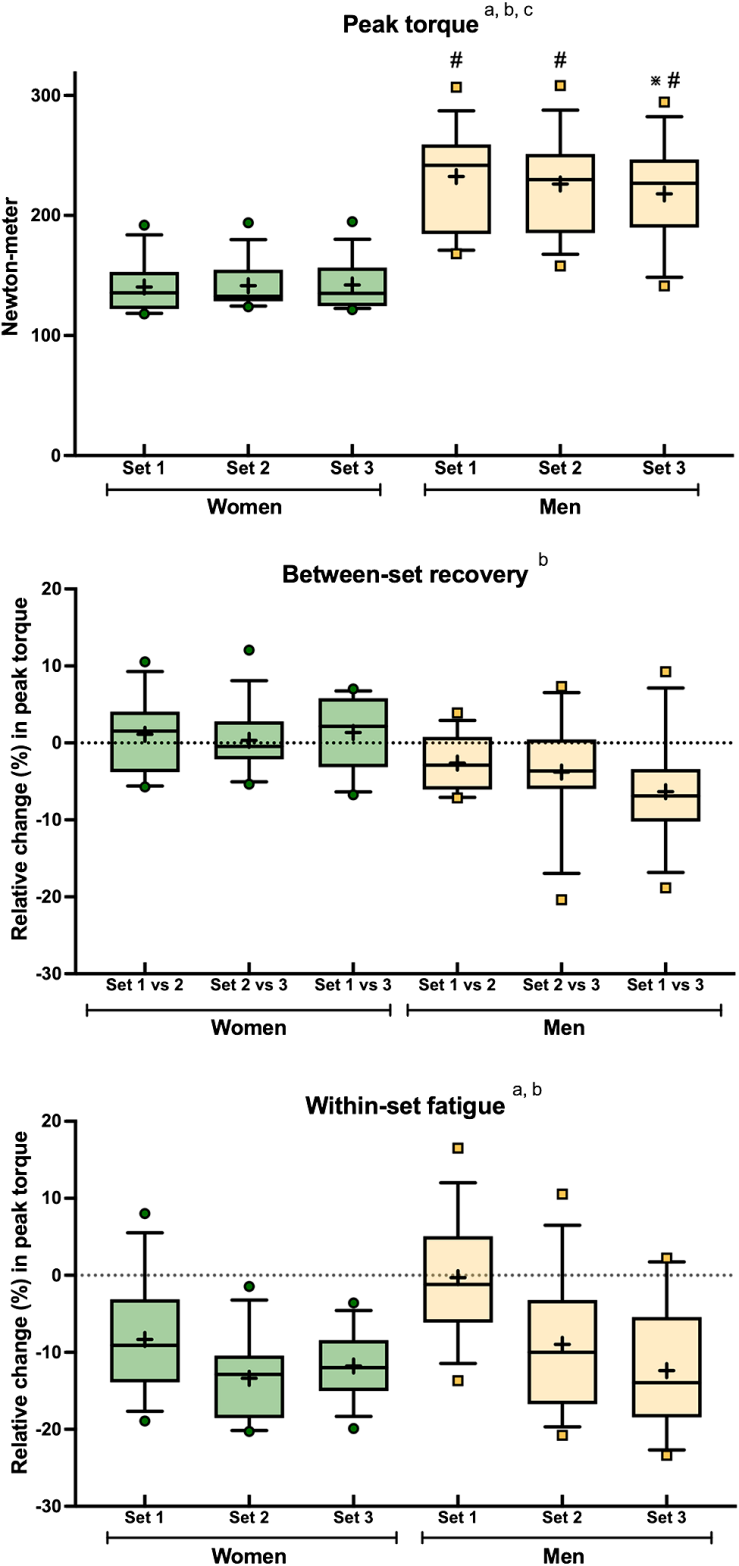
Performance, recovery, and fatigue variables from the isokinetic knee extension exercise for both women (green) and men (yellow). The boxes extend from the 25th to the 75th percentile, the line in the middle is the median, and the + symbol is the mean. The whiskers represent the 10th and 90th percentiles. a; main effect of set, b; main effect of sex (*P* = 0.056 if in grey), c; interaction set*sex. Post hocs: *Different with set 1 within a sex, #Different to women

Men produced higher absolute torque values than women, but they showed greater peak torque losses in both absolute (interaction set*sex *P* < 0.001; Fig. 3) and relative (main effect of sex *P* = 0.003, Fig. 3) values when compared with women. Within-set fatigue was also different between sexes (main effect of sex *P* = 0.034; Fig. 3). Here, however, the differences were mainly driven by the greater fatigue that women experienced within set 1 (~ 8% torque loss) compared to men (~ 0% torque loss). Our analysis also indicated that the greater within-set fatigue occurred in sets 2 and 3 independently of sex (main effect of set *P* < 0.001).

In general, there were significant correlations between within-set fatigue and between-set recovery only in the squat exercise in men (Supplementary Table 1).

## Discussion

We sought to comprehensively assess the relationship between aerobic capacity (V̇O_2max_) and various indices of recovery and fatigue during resistance exercise in men and women. In a secondary analysis, we also examined whether there were sex differences in recovery between sets and fatigue within sets. The results showed that aerobic capacity was weakly associated with between-set recovery and within-set fatigue during resistance exercise in both men and women. Women and men showed comparable levels of within-set fatigue in the multi-joint squat, but women showed more within-set fatigue during the single-joint isokinetic knee extension compared with men. In contrast, women recovered better than men between sets in both exercises.

The mixed-model ANOVA showed that V̇O_2max_ did not significantly covary with the different recovery and fatigue indices. It also showed that V̇O_2max_ did not significantly covary with the number of repetitions performed during the squat exercise. Although there were a few moderately strong correlations in the exploratory analysis, these must be interpreted with caution given the high number of correlations performed and the lack of association in the main mixed-model analysis. Our initial hypothesis that V̇O_2max_ would correlate with recovery between sets was based on the demonstrated relationship between aerobic capacity and recovery of PCr stores after strenuous exercise and repeated sprint performance [8, 9]. During recovery, the restoration of PCr generally follow a biphasic pattern, where more than half of the stores are restored within the first minute, while full restoration takes between 3 and 8 min [6, 8]. While the influence of aerobic capacity on recovery during resistance training has been scantly researched in the context of resistance training, it has been demonstrated that endurance trained athletes recover force faster following a 3-min rest period after 60% isometric contraction compared with power and strength athletes [18]. In addition, Ratamess reported that V̇O_2max_ significantly correlated with squat exercise performance (number of repetitions performed) across 1-, 2-, and 3-minutes recovery periods in resistance-trained men [10]. They concluded that aerobically fit individuals may not need as long rest intervals to maintain performance in the squat exercise as unfit individuals need. While our results appear to deviate to some extent from the Ratamess et al. study, we did find a correlation between V̇O_2max_ and the number of squat repetitions in set 3 in men (*r* = 0.604).The main differences between our study and Ratamess et al. were that V̇O_2max_ was determined by cycling in the present study, and running in Ratamess et al., and that we included both men and women in our study. However, both studies used the squats exercise involving multiple joints and relatively large muscle mass, and the correlations were trivial or moderate in both studies. The mean VO_2max_ was similar in our study and in the study by Ratamess et al. (48 ml/kg/min), but it should be remembered that our study included women, who had a slightly lower VO_2max_ than the men.

We postulate two candidates for the weak relationship between V̇O_2max_ and recovery. The first one is that the impact of aerobic power may become more important with repeated bouts/sets of high-intensity exercise. Indeed, previous studies have supported that V̇O_2max_ becomes increasingly important for performance after a few bouts of intense exercise [19]. This is also consistent with our finding of a correlation between V̇O_2max_ and the number of squat repetitions in set 3 in men. The second possibility is that there is an aerobic fitness threshold after which higher aerobic power will not lead to improved recovery between sets in resistance exercise. This hypothesis was raised already in 2001 by Tomlin and Wenger [8], who concluded that the lack of relationship between V̇O_2max_ and recovery from sprints in endurance runners might have been due to the already high V̇O_2max_ in these runners.

It has previously been shown that intense exercise involving large muscle mass displays a stronger relationship between V̇O_2max_ and PCr recovery than intense exercise using smaller muscle mass [8]. We found no correlation between V̇O_2max_ and isokinetic knee extension performance involving a smaller muscle mass than the squats. Similarly, V̇O_2max_ did not correlate with bench press performance in the Ratamess study. These findings may in part be related to the smaller muscle mass involved in bench press and the knee extension compared with squats, and are consistent with previous studies suggesting that multi-joint exercises involving large muscle mass may lead to almost double the acute oxygen consumption response compared with small muscle-mass exercises [20]. Even though individuals with high maximal aerobic power generally have increased concentrations of aerobic enzymes, increased mitochondrial number, size and surface area and increased myoglobin all contributing to improved oxygen extraction by muscle [21], it appears V̇O_2max_ may not fully reflect the oxidative capacity within the muscle, which is likely an important determinant of muscle recovery when using small muscle masses. Taken together, it seems likely that aerobic capacity is more important for recovery when multiple muscle groups are involved than isolated exercises involving single muscle groups.

In terms of fatigue within sets and recovery between sets, women and men showed comparable levels of within-set fatigue during the multi-joint squat, but women showed more within-set fatigue than men during the single-joint isokinetic knee extension. In contrast, women recovered better than men between sets in both exercises. Given the above results, we speculated that there would be a correlation between recovery between sets and fatigue within a set, i.e., that those who recovered their performance better between sets would also fatigue more within a set because they recovered more anaerobic phosphagen stores. However, this was generally not the case, although there were moderately strong correlations between fatigue within a set and recovery between sets for the men, but only for the squat exercise (Supplementary Table 1). Overall, the results suggest that men have a better PCr and/or glycolytic capacity that enables them to maintain high torque levels within relatively short sets of isolated exercise. This is supported by research showing that ATP, PCr and glycogen reductions are greater in men than in women after high-intensity exercise and repeated sprint exercise [22–24]. In contrast, the greater recovery between sets in women compared to men is likely due to differences in fiber and metabolic properties between the sexes, such as the greater area fraction of type 1 fibers in women than in men [25].

This study provided new information on the relationship between V̇O_2max_ and recovery and fatigue during two different resistance exercises. An obvious limitation is that we had no direct measurement of PCr resynthesis or other biochemical variables related to recovery or fatigue in skeletal muscle. In addition, we only tested a specific rest period (90 s for the squat and 45 s for the knee extension exercise). It is possible that V̇O_2max_ is more important for recovery between sets with different rest periods.

An attractive application of the current work is that resistance exercise design could potentially be tailored on an individual basis if typical strength and power losses are identified. For example, our results suggest that men may benefit from longer rest periods than women to restore power between sets. On the other hand, because women fatigue faster than men during isolated knee extensions within a set, it may be appropriate to prescribe different numbers of repetitions for men and women, particularly if explosive strength is the goal. This is supported by findings from velocity-based resistance training, where it has been reported that too many repetitions resulting in significant strength losses are negatively associated with the development of explosive strength [26, 27]. It should be acknowledged, however, that the individual variation within men and women is likely larger than the systematic difference between sexes. Thus, although there are some demonstrated sex differences in muscle fiber properties, such as a greater area fraction of type 1 fibers in women that may contribute to greater muscular fatigue resistance [28], using sex alone as the main variable for tailored program design seems far-fetched at this time.

## Conclusions

The findings revealed a weak association between aerobic capacity and between-set recovery and within-set fatigue during resistance exercise for both sexes. In terms of within-set fatigue, women and men demonstrated similar levels of fatigue in the multi-joint squat. However, women exhibited higher within-set fatigue during the single-joint isokinetic knee extension compared to men. Conversely, women displayed superior between-set recovery compared to men in both exercises. Overall, the current results suggest that aerobic capacity is not a critical factor in the recovery of performance between sets in resistance training. However, future studies should consider a broader range of resistance exercises, number of sets, and performance metrics to better inform personalized and evidence-based training prescriptions.

### Electronic supplementary material

Below is the link to the electronic supplementary material.

Supplementary Material 1

Supplementary Material 2

## Data Availability

Selected data are available upon reasonable request from the corresponding author.

## Abbreviations

ANOVA: Analysis of variance
V̇O_2max_: Maximal oxygen uptake
PCr: Phosphocreatine
RER: Respiratory exchange ratio
RPE: Rating of perceived exertion
1RM: 1 repetition maximum

## References

[1] de Salles BF, Simão R, Miranda F, Novaes Jda, Lemos S, Willardson A. Rest interval between sets in strength training. Sports Med Auckl NZ. 2009;39:765–77. 10.2165/11315230-000000000-00000 19691365

[2] Henselmans M, Schoenfeld BJ. The effect of inter-set rest intervals on resistance exercise-induced muscle hypertrophy. Sports Med Auckl NZ. 2014;44:1635–43. 10.1007/s40279-014-0228-0 25047853

[3] McKendry J, Pérez-López A, McLeod M, Luo D, Dent JR, Smeuninx B, et al. Short inter-set rest blunts resistance exercise-induced increases in myofibrillar protein synthesis and intracellular signalling in young males. Exp Physiol. 2016;101:866–82. 10.1113/EP085647 27126459

[4] Grgic J, Lazinica B, Mikulic P, Krieger JW, Schoenfeld BJ. The effects of short versus long inter-set rest intervals in resistance training on measures of muscle hypertrophy: a systematic review. Eur J Sport Sci. 2017;17:983–93. 10.1080/17461391.2017.1340524 28641044

[5] Tesch PA, Colliander EB, Kaiser P. Muscle metabolism during intense, heavy-resistance exercise. Eur J Appl Physiol. 1986;55:362–6. 10.1007/BF00422734 3758035

[6] Sahlin K, Harris RC, Hultman E. Resynthesis of creatine phosphate in human muscle after exercise in relation to intramuscular pH and availability of oxygen. Scand J Clin Lab Invest. 1979;39:551–8. 10.3109/00365517909108833 43580

[7] Yoshida T. The rate of phosphocreatine hydrolysis and resynthesis in exercising muscle in humans using 31P-MRS. J Physiol Anthropol Appl Human Sci. 2002;21:247–55. 10.2114/jpa.21.247 12491822

[8] Tomlin DL, Wenger HA. The relationship between aerobic fitness and recovery from high intensity intermittent exercise. Sports Med. 2001;31:1–11. 10.2165/00007256-200131010-00001 11219498

[9] Bogdanis GC, Nevill ME, Boobis LH, Lakomy HK. Contribution of phosphocreatine and aerobic metabolism to energy supply during repeated sprint exercise. J Appl Physiol Bethesda Md 1985. 1996;80:876–84. 10.1152/jappl.1996.80.3.876 8964751

[10] Ratamess NA, Rosenberg JG, Kang J, Sundberg S, Izer KA, Levowsky J, et al. Acute oxygen uptake and resistance exercise performance using different rest interval lengths: the influence of maximal aerobic capacity and exercise sequence. J Strength Cond Res. 2014;28:1875–88. 10.1519/JSC.0000000000000485 24714546

[11] Clark BC, Manini TM, Thé DJ, Doldo NA, Ploutz-Snyder LL. Gender differences in skeletal muscle fatigability are related to contraction type and EMG spectral compression. J Appl. 2003;94:2263–72. 10.1152/japplphysiol.00926.2002 12576411

[12] Nuzzo JL, Pinto MD, Nosaka K, Steele J. Maximal number of repetitions at percentages of the one repetition Maximum: a Meta-regression and moderator analysis of sex, Age, Training Status, and Exercise. Sports Med Auckl NZ. 2023. 10.1007/s40279-023-01937-7. 10.1007/s40279-023-01937-7 PMC10933212 37792272

[13] Hicks AL, Kent-Braun J, Ditor DS. Sex differences in human skeletal muscle fatigue. Exerc Sport Sci Rev. 2001;29:109–12. 10.1097/00003677-200107000-00004 11474957

[14] Hunter SK, Critchlow A, Shin I-S, Enoka RM. Fatigability of the elbow flexor muscles for a sustained submaximal contraction is similar in men and women matched for strength. J Appl Physiol. 2004;96:195–202. 10.1152/japplphysiol.00893.2003 14514707

[15] Hill EC, Housh TJ, Smith CM, Cochrane KC, Jenkins NDM, Cramer JT, et al. Effect of sex on torque, recovery, EMG, and MMG responses to fatigue. J Musculoskelet Neuronal Interact. 2016;16:310–7. PMC5259572 27973383

[16] Senefeld J, Pereira HM, Elliott N, Yoon T, Hunter SK. Sex differences in mechanisms of recovery after isometric and dynamic fatiguing tasks. Med Sci Sports Exerc. 2018;50:1070–83. 10.1249/MSS.0000000000001537 PMC5899026 29298217

[17] Gomes M, Santos P, Correia P, Pezarat-Correia P, Mendonca GV. Sex differences in muscle fatigue following isokinetic muscle contractions. Sci Rep. 2021;11:8141. 10.1038/s41598-021-87443-0 PMC8046769 33854136

[18] Häkkinen K, Myllylä E. Acute effects of muscle fatigue and recovery on force production and relaxation in endurance, power and strength athletes. J Sports Med Phys Fitness. 1990;30:5–12. 2195236

[19] Yoshida T, Watari H. Metabolic consequences of repeated exercise in long distance runners. Eur J Appl Physiol. 1993;67:261–5. 10.1007/BF00864226 8223541

[20] Farinatti PTV, Castinheiras Neto AG. The effect of between-set rest intervals on the oxygen uptake during and after resistance exercise sessions performed with large- and small-muscle mass. J Strength Cond Res. 2011;25:3181–90. 10.1519/JSC.0b013e318212e415 21993043

[21] Holloszy JO, Coyle EF. Adaptations of skeletal muscle to endurance exercise and their metabolic consequences. J Appl Physiol. 1984;56:831–8. 10.1152/jappl.1984.56.4.831 6373687

[22] Willcocks RJ, Williams CA, Barker AR, Fulford J, Armstrong N. Age- and sex-related differences in muscle phosphocreatine and oxygenation kinetics during high-intensity exercise in adolescents and adults. NMR Biomed. 2010;23:569–77. 10.1002/nbm.1495 20661873

[23] Esbjörnsson-Liljedahl M, Bodin K, Jansson E. Smaller muscle ATP reduction in women than in men by repeated bouts of sprint exercise. J Appl Physiol Bethesda Md 1985. 2002;93:1075–83. 10.1152/japplphysiol.00732.1999 12183505

[24] Esbjörnsson-Liljedahl M, Sundberg CJ, Norman B, Jansson E. Metabolic response in type I and type II muscle fibers during a 30-s cycle sprint in men and women. J Appl Physiol Bethesda Md 1985. 1999;87:1326–32. 10.1152/jappl.1999.87.4.1326 10517759

[25] Nuzzo JL. Sex differences in skeletal muscle fiber types: a meta-analysis. Clin Anat N Y N. 2024;37:81–91. 10.1002/ca.24091 37424380

[26] Pareja-Blanco F, Rodríguez-Rosell D, Sánchez-Medina L, Sanchis-Moysi J, Dorado C, Mora-Custodio R, et al. Effects of velocity loss during resistance training on athletic performance, strength gains and muscle adaptations. Scand J Med Sci Sports. 2017;27:724–35. 10.1111/sms.12678 27038416

[27] Pareja-Blanco F, Alcazar J, Sánchez-Valdepeñas J, Cornejo-Daza PJ, Piqueras-Sanchiz F, Mora-Vela R et al. Velocity loss as a critical variable determining the adaptations to Strength Training. Med Sci Sports Exerc. 2020;:1–1. 10.1249/MSS.0000000000002295 32049887

[28] Nuzzo JL. Narrative review of sex differences in muscle strength, endurance, activation, size, Fiber type, and Strength Training Participation Rates, preferences, motivations, injuries, and neuromuscular adaptations. J Strength Cond Res. 2023;37:494–536. 10.1519/JSC.0000000000004329 36696264

