# Supplementary material for: Relationship between maximal oxygen uptake, within-set fatigue and between-set recovery during resistance exercise in resistance-trained men and women
Source: BMC Sports Sci Med Rehabil. 2024 Feb 12;16:45. doi: 10.1186/s13102-024-00830-8 (PMC10863198; doi:10.1186/s13102-024-00830-8)
Supplement: Supplementary file 1 — Supplementary Material 1 [file 13102_2024_830_MOESM1_ESM.pdf]

Supplementary Questionnaire 1, Lundberg et al.

HEALTH DECLARATION

Date__________________ Date of birth ______________

Name____

Address____

Mailing address____

E-mail____

Phone home____________________ work____________________mobile_____________________

Height__________________ Weight_________________

Do you have, or have you previously had:	Yes	No	Comment
Asthma or other lung disease?	ÿ	ÿ	
Other allergic problems?	ÿ	ÿ	
Blood disease?	ÿ	ÿ	
Diabetes? Egg whites. sugar in the urine?	ÿ	ÿ	
Heart disease? Elevated blood pressure? Vascular	ÿ	ÿ	
disease?Joint or muscle disease? Back problems?	ÿ	ÿ	
Knee injury or knee problems? Other damage?	ÿ	ÿ	
Dizziness? Fainting? Epilepsy?	ÿ	ÿ	
Nose or throat infection	ÿ	ÿ	
Headaches (frequent, long-lasting or migraine)?		ÿ	
Do you take painkillers because of the pain?	ÿ	ÿ	
Other long lasting or serious illness/injury?Prescribed	ÿ	ÿ	
medications for long-term use?	ÿ	ÿ	
In addition, answer the following questions:			
Do you smoke? If yes, how much?	ÿ	ÿ	
Do you use snus? If yes, how much?	ÿ	ÿ	
Do you use any medication regularly?	ÿ	ÿ	

Do you take any food supplement?	ÿ	ÿ	___________________________________
Do you feel completely healthy right now?	ÿ	ÿ	___________________________________

I certify that the above information has been truthfully entered by me:

Signature____

Any additional comments:

____

____

____

____

____

Thank you for your participation!
